# Brugada syndrome in the forensic field: what do we know to date?

**DOI:** 10.3389/fcvm.2025.1618762

**Published:** 2025-08-11

**Authors:** Oscar Campuzano, Simone Grassi, Estefanía Martínez-Barrios, Andrea Greco, Vincenzo Arena, Georgia Sarquella-Brugada, Antonio Oliva

**Affiliations:** ^1^ Medical Science Department, School of Medicine, University of Girona, Girona, Spain; ^2^ Institut Investigacions Biomèdiques de Girona, IDIBGI, Girona, Spain; ^3^ Centro de Investigación Biomédica en Red, Enfermedades Cardiovasculares, Madrid, Spain; ^4^ Department of Health Sciences, Section of Forensic Medical Sciences, University of Florence, Florence, Italy; ^5^ Pediatric Arrhythmias, Inherited Cardiac Diseases and Sudden Death Unit, Cardiology Department, Sant Joan de Déu Hospital de Barcelona, Barcelona, Spain; ^6^ Arrítmies Pediàtriques, Cardiologia Genètica I Mort Sobtada, Malalties Cardiovasculars en el Desenvolupament, Institut de Recerca Sant Joan de Déu, Esplugues de Llobregat, Barcelona, Spain; ^7^ European Reference Network for Rare, Low Prevalence and Complex Diseases of the Heart (ERN GUARD-Heart), Amsterdam, Netherlands; ^8^ Area of Pathology, Department of Woman and Child Health and Public Health, Fondazione Policlinico Universitario A. Gemelli IRCCS, Rome, Italy; ^9^ Pediatrics Department, School of Medicine, Universitat de Barcelona, Barcelona, Spain; ^10^ Institute of Public Health, Section Legal Medicine, Catholic University, Rome, Italy

**Keywords:** sudden cardiac death, channelopathies, Brugada syndrome, forensics, molecular autopsy, structural alterations

## Abstract

Brugada Syndrome is a cardiac genetic entity associated with an elevated risk of life-threatening arrhythmias, making accurate and prompt diagnosis vital to prevent lethal outcomes. To date, no macroscopic alterations have been identified in diagnosed patients, but microscopic alterations have been reported in some cases, which remain a matter of argue. This is especially relevant in the forensic field, helping to perform a post-mortem diagnose. Molecular autopsy may help to identify the genetic alteration, but other data such as family history and the situation of death are crucial to unravel the definite cause of an unexpected decease. Deleterious variants in the *SCN5A* gene are the most common cause of Brugada syndrome; however, the genetic diagnostic yield of Brugada Syndrome remains low, with a deleterious variant in *SCN5A* identified in only a 25%–30% of cases, and a high number of phenotype-positive genotype-negative individuals. This along with a proper clinical-genetic interpretation and the management of variants of unknown clinical significance remains a current challenge. Our review aims to update the available forensic data focused on autopsies performed in Brugada syndrome cases.

## 1 Introduction

Brugada syndrome (BrS) is a genetic cardiac ion channel entity that can lead to malignant arrhythmias and sudden cardiac death (SCD). It accounts for nearly 5% of all SCD and almost 20% of SCD that occur in the absence of structural cardiac abnormality (1). Lethal episodes should occur mainly at rest/during the night, usually in males nearly 40 years old but it may also be diagnosed in children. BrS is considered to cause 10%–20% of sudden infant deaths and 4%–12% of SCD in children (2). The estimated prevalence of BrS is approximately 1:5000, and it is 8–10 times more common in males than in females. In Southeast Asia is widely reported a highest prevalence of BrS, up to 14 times, being the primary cause of natural death among males below the age of 50. In this region, the condition is often referred to as sudden unexpected nocturnal death syndrome (SUNDS), given its predilection for causing death during sleep.

The clinical diagnosis of BrS is based on the typical electrocardiogram (ECG) called type 1 and characterized by a coved-type ST-segment elevations in the right precordial leads (V1–V3) (3). This ECG pattern is often dynamic and may appear spontaneously (basal situations) or be unmasked (using sodium channel blockers or external triggers such as fever). Symptoms may range from asymptomatic to ventricular fibrillation, syncope and even SCD as the first manifestation of the disease (4). Given the potential severity of the disease, early identification is crucial to implement personalized risk reduction strategies. Current recommendations support implantable cardioverter-defibrillator (ICD) therapy in BrS patients with arrhythmogenic syncope or documented ventricular arrhythmias. However, asymptomatic patients should receipt a close follow-up despite low risk of lethal episodes (5).

BrS represents a relatively rare but lethal event, having significant consequences not only in the clinical setting (particularly in the screening of first-degree relatives) but also in the forensic field, as a portion of sudden unexpected deaths remain unsolved after a comprehensive medico-legal autopsy could potentially be explained by BrS.

## 2 Genetic diagnostics

**Figure 1:**
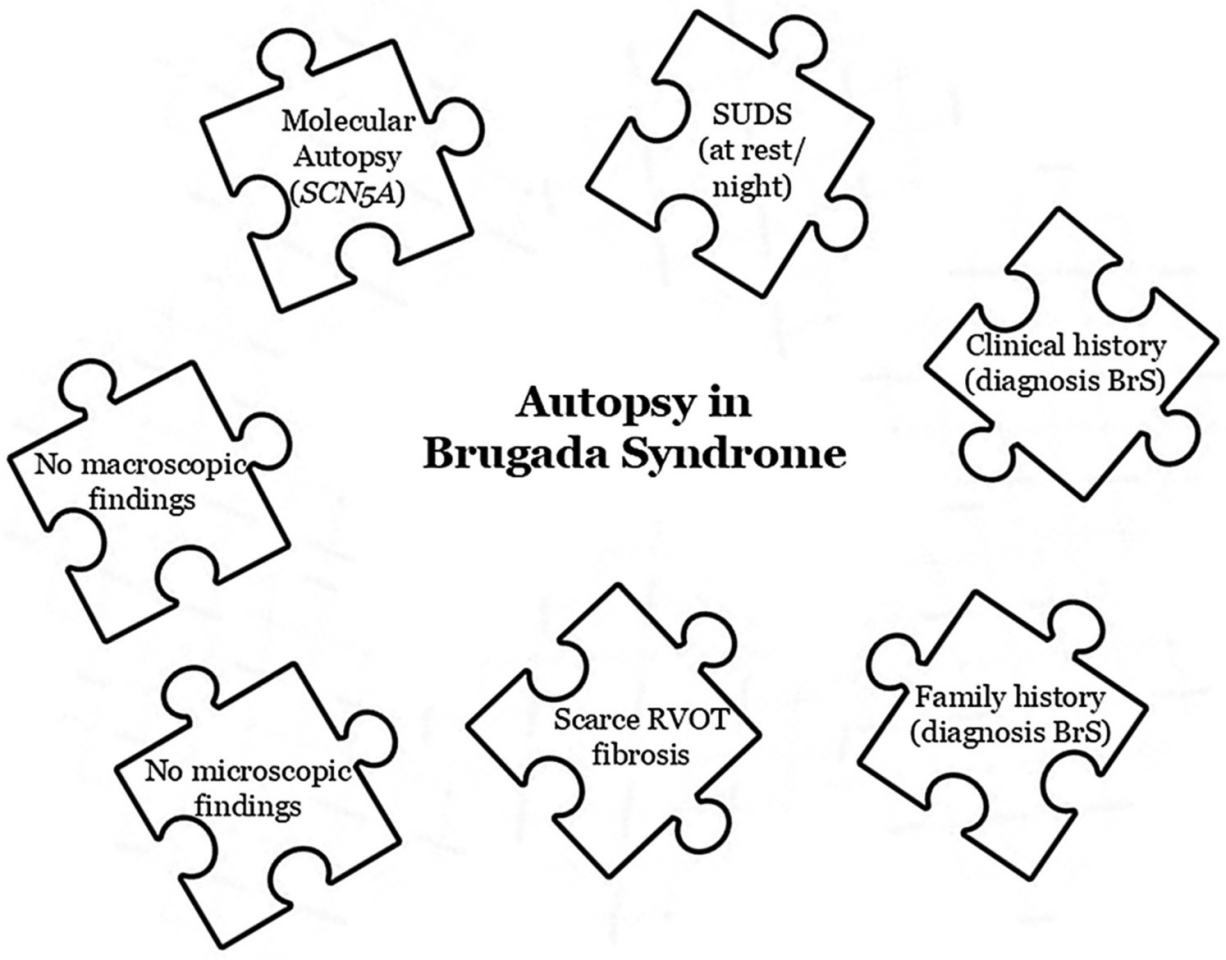
Key pieces in a Brugada syndrome's autopsy.

Today, genetic testing is a highly relevant tool in the definitive diagnosis of a cardiac channelopathy such as BrS. Current guidelines recommend performing a genetic test when a patient is diagnosed with BrS or when there is clinical suspicion (Figure 1). This genetic study should also be performed postmortem as part of a complete autopsy procedure (molecular autopsy) particularly if the death meets the criteria for suspected BrS or if there are individuals in the family diagnosed with BrS, with or without symptoms.

Genetic tests for BrS can include various genes with potential association with the disease, although only rare variants located in the *SCN5A* gene are currently definitively linked to its pathogenesis (3). This gene, located on chromosome 3p21, encodes the voltage-dependent sodium cardiac channel subunit (Nav1.5). Likely pathogenic or pathogenic (LP/P) variants have been identified in families with an autosomal dominant pattern of inheritance. These genetic alterations result in a loss-of-function of the Nav1.5 channel. Nowadays, a conclusive genetic alteration is identified in 25%–30% of families with a clinical diagnose of BrS. Additionally, also in the *SCN5A* have been reported gross insertions/deletions (Copy Number Variants, CNV) despite punctual studies performed so far suggest that less than 2% of BrS cases may be associated with these deleterious genetic alterations. Other minor genes encoding mainly sodium subunits or associated proteins have been proposed as potential causes of BrS, however further studies are needed to determine their definite role (6). Concerning pattern of inheritance, today only autosomic dominant is widely accepted; other patterns such as autosomic recessive, X-linked or even mitochondrial have been suggested but no definitively accepted so far.

Taking all these data, at this point is important to remark that the main fact is perform a thorough interpretation and a conclusive final classification following the American College of Medical Genetics and Genomics and the Association for Molecular Pathology (ACMG/AMP) guidelines (7) are required for any variant to be considered LP/P, thus confirming the genetic basis of BrS (3).

Currently, in up to 40% of sudden death cases, a conclusive cause of death after a comprehensive medico-legal autopsy remains to clarify (8, 9). In such cases, the term “negative autopsy” is used, which broadly includes both the absence of cardiac anomalies and the presence of nonspecific findings that do not clearly indicate a precise cause of sudden death. Molecular autopsy refers to a post-mortem genetic analysis or post-mortem molecular analysis used in forensic medicine, focused on the application of genetic diagnostic in post-mortem samples (10–12). Therefore, in cases of previously diagnosed BrS or suspected BrS as the cause of an unexpected decease, molecular autopsy has become a fundamental tool in the current forensic area as an essential complement to standard medico-legal autopsy protocols (13).

A significant portion of rare variants detected in genetic studies related to cardiac channelopathies are classified as having ambiguous significance (variants of unknown significance, VUS). This is also common in BrS, especially following a molecular autopsy. These VUS cannot be clinically actionable because we do not know the role they may play in the origin and/or progression of BrS. However, they cannot be dismissed entirely due to their ambiguous nature and lack of information that would allow us to classify them with a definitive role (3). In particular, in the forensic context, information like familiar/personal anamnesis and segregation analysis are often missing/incomplete, and thus VUS represent a frequent issue when the cause of the death must be assessed (14). VUS should be reinterpreted periodically, in light of the ongoing updates in clinical and genetic databases. Our recent research suggests reclassifying such variants within no more than five years after their initial classification, with a minimum interval of two years (15, 16). This interval may vary depending on the available resources of each center, although we recommend at least performing an update of the variant frequencies in the population annually, given the ease of the process and the rapid evolution of the available datasets.

## 3 Structural findings

To date, BrS is classified as an inherited cardiac channelopathies leading to malignant arrhythmias in a structurally normal heart (Figure 1). From a pathological perspective, it is widely accepted that no morphological heart alterations are present at the macroscopic level. Nevertheless, many reported forensic cases continuous to associated the with microscopic anomalies that *per se* have uncertain significance (17). Therefore, autopsies in BrS typically reveal no consistent structural abnormalities and are generally reported as negative. However, emerging evidence suggests the presence of structural alterations in the hearts of some BrS patients (18, 19) despite no conclusive origin of these scattered alterations (cause or consequence of the arrythmia) have been clarified to date. In a comprehensive study performed by our group, we identified that no definite diagnosis of BrS was reported in some cases presenting microscopic structural alterations (20).

Current evidence on BrS-related structural changes mainly relies on clinical endomyocardial biopsies, explanted heart’ samples, post-mortem microscopic data and radiological images (in particular obtained through cardiac magnetic resonance, that represents the gold standard for right ventricle and replacement fibrosis imaging) (21). Many histological anomalies have been described in BrS cases, with the most recurring ones represented by signs of acute or chronic myocarditis (inflammatory infiltrates with myocardial necrosis, interstitial fibrosis, epi-to-endocardium fibrosis) and fatty infiltration (20). These findings mainly affect the anterior part of the right ventricle outflow tract (RVOT), with inflammation and fibrosis being the only changes statistically associated with BrS (22). A correlation between some structural changes (namely, fibrosis and decreased gap junction expression) and electrical anomalies in the RVOT has been described, with these alterations thought to be primarily responsible for conduction velocity anomalies (low-voltage areas) (23, 24). The reason for the predominant involvement of RVOT is not fully understood, but it may be related to an abnormality in the migration of cardiac neural crest cells, that express connexin-43 (whose expression -as said- is often reduced in BrS patients’ RVOT) and are involved in the development of the RVOT. Structural changes in BrS are not static: Isbister et al. performed periodic cardiac magnetic resonances on BrS patients, finding that they tended to develop focal fibrosis in the right ventricle side of the basal spectrum, failing to find diffuse myocardial fibrosis at the parametric mapping (21).

In BrS, the RVOT dilation has also been found to be more frequent in carriers of *SCN5A* pathogenic variants (25, 26). In addition, fibrosis in BrS is not exclusive to the RVOT, having been also found in the epicardial surface of the left ventricle, especially in carriers of pathogenic *SCN5A* variants (27). All data available so far suggests a careful microscopic heart analysis during autopsy of diagnosed or suspected BrS cases, especially areas of the RVOT. This is also important given the existence and frequency of the so-called Brugada clinical phenocopies (i.e., other disorders that produce a Brugada-like ECG pattern), like some cases of right or left ventricular hypertrophy or tricuspid valve defect (28). Moreover, BrS cases frequently tend to show anomalies suggestive of the first stages of arrhythmogenic cardiomyopathy (ACM), like fibro-fatty myocardium replacement and RVOT dilation (29), that are also associated with a higher risk of severe ventricular arrhythmias (30). Moreover, a correlation between *SCN5A* variants and ACM has been reported, which could be explained by the influence of an underexpression in sodium channels over proteins that interact with Nav1.5 in the intercalated disks (31). For these reasons, the idea of a BrS-arrhythmogenic cardiomyopathy overlapping syndrome is preferred over the concept of the cardiomyopathy as a BrS phenocopy (32). In these cases, genetic testing plays a pivotal role, as it can contribute to achieving a precise diagnosis and uncover potential overlapping syndromes.

The relationship between genotype and histotype is an actual frontier in this field of research. While a statistically significant association between fibrosis and *SCN5A* variants in clinical BrS patients has not yet been firmly established, studies in *SCN5A* knockout mice show that the extent of fibrosis is greater than in the wild-type and that it tends to increase with age, particularly in males (33). Overall, structural anomalies at the histological and radiological examination tend to be more frequent in carriers of *SCN5A* pathogenic variants (25). The integration of microscopic and genetic information is particularly important in forensic settings, since the high prevalence of pathogenic *SCN5A* variants in young victims of BrS arrhythmias tends to suggest that SCD in BrS is substantially of electric nature in the young age, while a structural contribution mainly concerns the SCD in adult age, that represent most of the event's (31). Finally, it is important to remark that, from our point of view, no case with a definitive diagnosis of BrS has progressed to the definitive diagnosis of any cardiomyopathy but this fact does not imply that the identification of microscopic heart anomalies justify the exclusion of BrS as a possible diagnosis.

## 4 Family role

The sudden death of an individual has a devastating impact on family members. This situation is exacerbated when the suddenly deceased relative is young, or even a child. Such situations often occur in sudden deaths related to BrS, where the autopsy usually fails to identify any findings that explain the cause of death. This leaves families anguished, without answers regarding the cause of such a lethal event. In 2021, Alghamdi et al. coined the term “molecular autopsy by proxy” to describe the genetic testing conducted on family members of deceased relatives with the purpose of determining the underlying cause of their death (34).

Current clinical guidelines recommend conducting a clinical assessment of all family members when a sudden and unexplained death occurs, because cardiac channelopathies, such as BrS (Figure 1), are usually the primary suspect cause of decease (35, 36). The main objective of genetic screening in relatives is to identify these conditions to potentially prevent malignant arrhythmias in family members. Evaluation of families can identify an inherited cardiac condition in one-quarter of previously unsuspected relatives, including BrS (37, 38). Systematic use of sodium-blacker testing with high right precordial ECG leads up to 3-fold increase the diagnostic yield of BrS cases (39, 40). Molecular autopsy is recommended in these cases, as abovementioned but, if not performed for any reason, genetic analysis is any of clinically diagnosed or highly suspected relatives should be done (41). However, the diagnostic yield is low which coupled with a current genetic yield in BrS of nearly 30% would significantly underestimate its significance (3). Nowadays, it is recommended that genetic testing of family members should only be performed if a LP/P variant has been identified in the decedent; our experience suggest to perform cascade genetic analysis of VUS variants, as our results show that a large proportion of VUS can be eliminated as a primary cause of BrS, as we found clinically diagnosed relatives who are not carriers of the rare variant. We emphasize that VUS should not be transferred to clinical practice or be clinically actionable, but genetic segregation can rule out the detrimental role of a large proportion of VUS, reducing the anxiety of being a carrier of a genetic alteration with no clear role in BrS.

## 5 Conclusions

BrS cases are often considered completely “silent” at the autopsy. Today, several elements (including situation of death, clinical family history, macroscopic/microscopic findings, molecular autopsy) should be taken into account to support a conclusion of BrS as the cause of an unexpected and unexplained death. To date no case with a definitive diagnosis of BrS has progressed to the definitive diagnosis of any cardiomyopathy, reinforcing BrS as a pure ion channel disease without structural heart alterations. However, this does not mean that the presence of microscopic cardiac anomalies should exclude BrS as a potential diagnosis despite the need to clarify if these scattered tissular alterations are the cause or may be a consequence of the malignant arrhythmia.
